# Cutaneous biodistribution of topically applied active ingredients: Evaluation of intra- and inter-laboratory reproducibility

**DOI:** 10.1016/j.ijpx.2026.100618

**Published:** 2026-07-16

**Authors:** Si Gou, Anne Moustie, Kevin Caché, Luca Insolia, Jun Wu, Sébastien Grégoire, Stéphane Guerrier, Yogeshvar N. Kalia

**Affiliations:** a School of Pharmaceutical Sciences, University of Geneva, 1211 Geneva, Switzerland; b Institute of Pharmaceutical Sciences of Western Switzerland, University of Geneva, 1211 Geneva, Switzerland; c L'Oréal R&I France, 1 Av. Eugène Schueller, 93600 Aulnay-Sous-Bois, France; d Department of Earth Sciences, University of Geneva, 1206 Geneva, Switzerland; e Geneva School of Economics and Management, University of Geneva, 1211 Geneva, Switzerland

**Keywords:** Cutaneous biodistribution method, Intra-laboratory, Inter-laboratory, Validation, Mathematical model, Topically applied active ingredients

## Abstract

A robust method to quantify the spatial distribution of active ingredients and their concentrations as a function of depth within the epidermis and dermis would be advantageous for both cosmetic and pharmaceutical products. Here, a cassette-based in vitro permeation test (IVPT) was performed (at a given site) under finite dose conditions with five physicochemically distinct substances (with a > 350,000-fold difference in lipophilicity) using human abdominal skin – both permeation and total deposition were determined. A total of eighteen skin discs (area = 2 cm^2^, 6 female donors, 3 samples per donor) were used for the IVPT. They were subsequently divided into three groups and distributed to L'Oréal Operator 1 (L1, *n* = 6), L'Oréal Operator 2 (L2, n = 6), and University of Geneva (UG, n = 6). The cutaneous biodistribution method was then used to determine the spatial distribution of the five substances at a high resolution from the stratum corneum down to the upper dermis. Twenty skin lamellae with a thickness of 20 μm were obtained from each disc and the amount of each substance in each lamella was quantified. A novel statistical analysis was employed to investigate the variability/reproducibility of the data generated intra-laboratory (L1 vs. L2) and inter-laboratory (L1 vs. UG and L2 vs. UG). The results demonstrated the absence of significant difference in the permeation, total deposition, and cutaneous biodistribution data obtained following the identical protocols at both the intra- and inter-laboratory level and confirmed the robustness of the cutaneous biodistribution method in understanding the disposition of topically applied substances in the skin.

## Graphical abstract

**Figure:**
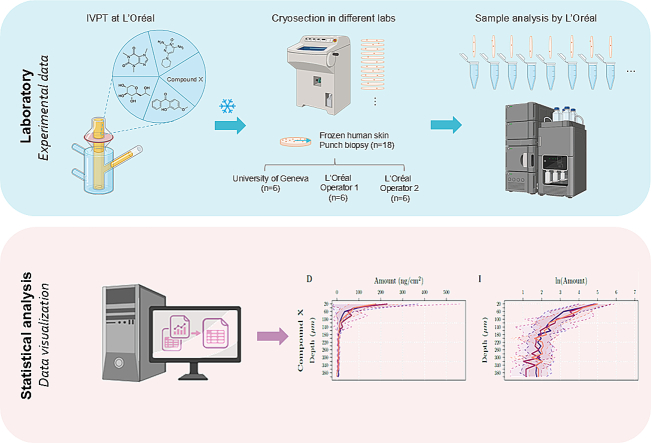

## 1 Introduction

The majority of topically applied active pharmaceutical ingredients (APIs) for the management of dermatological symptoms are normally intended to penetrate through the stratum corneum (SC) to reach their targets in the viable epidermis and dermis, with limited systemic absorption. Similarly, the target site of cosmetic ingredients can also vary – i.e. moisturizers are designed to remain in the stratum corneum, and their penetration across the skin barrier is not desired and should be prevented. In contrast, for antioxidant activity, depigmentation, skin rejuvenation and hair growth, the outcome may rely on the amount of active ingredient penetrated through the stratum corneum and into the viable epidermis and hair follicles. Therefore, in addition to the quantification of total deposition in the skin, it is of great interest to assess the spatial distribution of active ingredients as a function of penetration depth for the development of safe and effective formulations for both pharmaceutical and cosmetic indications.

The in vitro permeation test (IVPT) can be used in combination with tape stripping to investigate skin penetration, cutaneous bioavailability, or the pharmacokinetics of topically applied substances (Lane, 2024). In this method, successive layers of the stratum corneum are progressively removed by using adhesive films upon the completion of an in vitro permeation study and the amount of the penetrated formulation can be determined by using different analytical measurements (Marttin et al., 1996; Mohammed et al., 2012; Mendelsohn et al., 2006; Mao et al., 2012; Pirot et al., 1997). However, the quantity of stratum corneum removed in each tape strip can be significantly altered due to multiple factors, including skin hydration, vehicle/ formulation, the type of adhesive tape, the pressure with which the tape is applied to the skin, and the velocity of its removal. Weighing tape-strips to accurately determine the quantity of stratum corneum removed in each strip and hence estimate the approximate thickness of membrane that has been removed is not adapted to routine use (Kalia et al., 1996). Alternative indirect methods such as colorimetric methods for protein quantification (Dreher et al., 1998), and UV–Vis absorption (Jacobi et al., 2005), are also time consuming and can lack specificity. Another drawback of the tape stripping method is that it only measures the amount of substance in the stratum corneum and there is no direct drug quantification in the viable epidermis and dermis.

It would be advantageous to measure the concentrations, present in the different anatomical regions of the skin, and also to know the spatial distribution profile as a function of penetration depth, which can also be determined as a function of time. The “cutaneous biodistribution method” (CBM) enables the determination of the concentration of a penetrant as a function of depth within the skin, providing insight into the spatial distribution of active ingredients and formulation excipients. It has been used to determine the biodistribution profile of drug molecules with different physicochemical properties and therapeutic indications, including tacrolimus (Lapteva et al., 2014), aciclovir (Chen et al., 2016), imiquimod (Lapteva et al., 2019), vismodegib (Kandekar et al., 2019), sirolimus (Quartier et al., 2021), spironolactone (Dahmana et al., 2021), amorolfine/econazole/terbinafine (Gou et al., 2022) and cannabidiol (Lapteva et al., 2024). Briefly, upon completion of an in vitro permeation study following standard experimental protocols, a skin disc (typically with an area of 0.5–0.8 cm^2^) is punched out of the skin sample and snap frozen. A series of lamellae with predetermined thickness (e.g. 20 lamellae each with a thickness of 20 μm), descending from the skin surface to the upper dermis can be generated by cryosectioning. Subsequent extraction of the molecule of interest from each lamella is followed by quantification using a high sensitivity method, such as ultra high-pressure liquid chromatography (UHPLC) coupled with tandem quadrupole mass spectrometry (MS/MS). This methodology is also of interest with respect to the topical administration of cosmeceuticals where detailed information on the localisation of formulation components could improve the assessment of safety since it would be possible to determine the concentrations of substances as a function of position. However, the application of this methodology more generally requires further validation in terms of reproducibility between operators either intra- or inter-laboratory.

The objective of this work was to evaluate the inter-operator and inter-site reproducibility of the cryosectioning steps of the CBM. To realise the objective, a detailed investigation into the cutaneous delivery of five active compounds was performed with the in vitro permeation test (IVPT) experiments carried out at a single site – L'Oréal – and the cryosectioning procedure being performed by the three different operators (two at L'Oréal and one at the University of Geneva). Since the IVPT studies and the quantification were done at a single site, any variability would be due to the cryosectioning step.

The five compounds of interest had varied physicochemical properties including a > 350,000-fold difference in lipophilicity (Proxylane™, Hydroxypropyl tetrahydropyrantriol; Caffeine, 1,3,7-triméthylxanthine; Minoxidil™, 6-(1-Piperidinyl)-2,4-pyrimidinediamine 3-oxide; Oxybenzone, 2-Hydroxy-4-methoxy-benzophenone; Compound X, a proprietary representative compound chosen for its physicochemical characteristics, notably its log P, to complete the lipophilicity range in order to cover a large number of cosmetic products) (Table 1). A cassette-based dosing approach involving simultaneous application of the five molecules with a wide range of log P (−1.51 to 4) from a single formulation under finite dose conditions was used. Preliminary studies were performed to ensure that caffeine, minoxidil, compound X and oxybenzone could be quantified with a common analytical method and that all chemicals could be efficiently extracted using the same extraction protocol.

**Table 1:** Physicochemical properties of the investigated active ingredients.

Compound	Proxylane™	Caffeine	Minoxidil™	Compound X	Oxybenzone
Structure				Confidential	
M.W.	192	194	209	194	228
Log_10_P (exp)	−1.51	−0.07	0.87	1.6	4.03
pKa’	/	/	4.61 (AH/A-)	11.2 (AH/A-)	7.6 (AH/A-)
pKa”				9.5 (AH/A-)	

Therefore, the specific aims of the study were (i) to develop highly selective and specific analytical methods for the quantification of the five active ingredients at the nanogramme level, (ii) to evaluate the in vitro permeation and total skin deposition of the five active ingredients. (iii) to analyse the spatial biodistribution of the five substances in the skin as a function of depth, and thereby (iv) to validate the reproducibility of the CBM at the intra- and inter-laboratory level by comparing the biodistribution profile generated by three individual operators at two different laboratories using a robust statistical methodology.

## 2 Material and methods

### 2.1 Materials

Proxylane™ (CAS 439685–79-7), deuterated proxylane, Minoxidil™ (CAS 38304–91-5), oxybenzone (CAS 131–57-7), and compound X were provided by L'Oréal. Caffeine (CAS 58–08-2), caffeine trimethyl-13C3 (CAS 78072–66-9), oxybenzone phenyl deuterated D5 (CAS 1219798–54-5) were purchased from Sigma Aldrich (Lyon, France). Methanol and acetonitrile (LC-MS grade) were purchased from Carlo Erba (France), formic acid (extra pure 99%) was purchased from VWR (Germany). Deionized water was obtained from the MilliQ IQ 7003 system. Sodium chloride and Tween 80 reagents were purchased from Fisher (Belgium).

### 2.2 Analytical method

A UHPLC-MS/MS method using a binary solvent pump Nexera Shimadzu LC system coupled with an API3500 triple quadrupole mass spectrometer from Sciex was developed to quantify cutaneous permeation, total skin deposition and biodistribution of proxylane, minoxidil, oxybenzone, compound X, and caffeine. Deuterated proxylane, oxybenzone phenyl deuterated D5, caffeine trimethyl-^13^C3 were used as internal standards. No internal standard was used for compound X and minoxidil.

Chromatographic separation for oxybenzone, minoxidil, caffeine, and compound X was performed under gradient condition 1 (Table 2) on a Supelco Ascentis C18 column (50 × 2.1 mm, 2.7 μm) and the column temperature maintained at 50 °C. The mobile phase consisted of methanol:0.1% formic acid (50:50, *v*/v) with a flow rate at 0.8 mL/min. Chromatographic separation for proxylane was performed under gradient condition 2 (Table 2) on a Phenomenex Luna NH_2_ column (150 × 4.6 mm, 3 μm) and the column temperature maintained at 50 °C. The mobile phase consisted of methanol:ammonium formate (100 mM, pH = 3.2) (50:50, *v*/v) with a flow rate at 1.2 mL/min. The injection volume was 5 μL.

**Table 2:** Flow rate and mobile phase composition in the gradient separation method.

Gradient condition 1^a^				Gradient condition 2^b^			
Time (min)	Flow rate (mL/min)	%A	%B	Time (min)	Flow rate (mL/min)	%A	%B
0	0.8	100	0	0	1.2	0	100
0.5		100	0	2		10	90
2		0	100	2.01		100	0
3		0	100	3		100	0
3.01		100	0	3.01		0	100
4		100	0	4.5		0	100

a For oxybenzone, minoxidil, caffeine, and compound X.

b For proxylane.

Nitrogen was used as the drying gas and collision gas, respectively. The detection was performed with electrospray ionization (ESI) in a switch positive/negative mode for oxybenzone, minoxidil, caffeine, and compound X, while in a negative mode for proxylane using multiple reaction monitoring (MRM). The mass transition for the parent to product ion [M-H^+^] for each analyte and the other MS/MS settings, including declustering potential, entrance potential, collision energy and cell exit potential are reported in Table 3. Data acquisition and processing were carried out using Analyst® software Version 1.7.2. The UHPLC-MS/MS method was validated according to *Bioanalytical FDA guidelines* and details are provided in the Supplementary Information (Fig. S1-S2 and Tables S1-S5).

**Table 3:** The MS/MS settings for the detection of the investigated active ingredients and their internal standards.

Compound	Proxylane	Deuterated proxylane	Caffeine	Caffeine trimethyl-^13^C3	Minoxidil	Compound X	Oxybenzone	Oxybenzone phenyl deuterated D5
Q1	237	243	195	198	210	192.9	229	234
Q3	45.1	45.1	138.1	140	164	174.9	151	151
Dwell	150	150	30	30	30	40	30	30
Declustering Potential (V)	−35	−35	30	30	90	−65	85	85
Entrance Potential (V)	−10	−10	7	7	7	−10	4	4
Collision Energy (V)	−10	−10	26	26	32	−23	24	24
Cell Exit Potential (V)	−10	−10	10	10	5	−10	11	11
Source temp	700 °C							
Curtain gas	25 Psi							
Gas 1	55 Psi							
Gas 2	40 Psi							
CAD	4 Psi							
Capillary voltage	+5500 V (ESI+)− 4500 V (ESI−)							

### 2.3 Skin preparation

Frozen human abdominal skin was obtained from the Les Princes clinic, Biopredic and Alphenyx companies, and were obtained from anonymous healthy female donors during plastic surgery procedures according to the French regulations (article L.1243–4 of the French public Health Code) and Declaration of Helsinki act. Patients' written informed consents were collected and kept by the surgeon. Only age, sex and anatomical site of samples were reported. After the surgery, the skin was stored at 4 °C, then the hypodermis and adipose tissue were carefully removed from the fresh skin within a maximum of 5 days. The skin was then stored at −20 °C and delivered frozen to L'Oréal. The excised skin samples were horizontally sliced with an Acculan 3Ti dermatome GA670 (Aesculap, B.Braun, Tuttlingen, Germany) to a nominal thickness of 1100 μm. Then, skin samples were extemporaneously punched out into 32 mm diameter circular discs for the in vitro skin delivery studies.

### 2.4 In vitro skin delivery studies

The IVPT studies were performed at L'Oréal (by L'Oréal Operator 1 and L'Oréal Operator 2) following the OECD Test Guideline 428 using standard Franz diffusion cells (area = 2 cm^2^). Frozen dermatomed human abdominal skin samples (*n* = 18, thickness = 927 ± 99.6 μm) generated from 6 female donors were thawed at room temperature for 30 min and were then mounted on diffusion cells, the donor and receptor compartments were filled with buffered saline (0.9%, *w*/*v*) containing 0.25% Tween 80. The solubility of the investigated active ingredients in the fresh receptor fluid was evaluated previously and details are provided in the Supplementary Information (Table S6). After equilibration for 60 min, skin integrity was monitored by measuring the Transepidermal Water Loss (TEWL) with Delfin© evaporimeter. Only skin samples with TEWL <20 g/m^2^/h were employed for the skin delivery study (mean TEWL was 14.8 ± 2.9 g/m^2^/h, with individual values ranging from 12.1 to 19.7 g/m^2^/h).

A solution of the cassette formulation (a mixture of propylene glycol/ethanol/water, 5/46.65/46.65, *w*/w/w) containing caffeine (0.25%, *w*/*v*), proxylane in solution at approximately 35% in a mixture of water and 1,2-propane diol 60/40 (0.25%, w/v), minoxidil (0.25%, w/v), compound X (0.25%, w/v) and oxybenzone (0.25%, w/v) was applied to the skin surface (5 mg formulation/cm^2^ – respecting finite dose conditions). The receptor compartment was stirred and maintained at 32 ± 1 °C throughout the experiment for 16 h. At the end of the experiment, the residual formulation was removed from the skin surface by washing the skin twice with 0.6 mL of 5% lauryl ether sulphate, rinsing thrice with water, and drying with cotton swabs. Diffusion cells were dismantled and 1.5 mL of the receptor phase were withdrawn to analyse the amount of each substance permeated across the skin using the validated UHPLC-MS/MS method.

### 2.5 Biodistribution of the investigated active ingredients in human skin

**Fig. 1:**
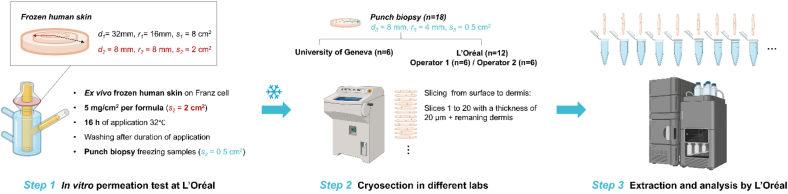
Schematic illustration of the study design and protocol for the skin samples assigned to L'Oréal Laboratories (Operator 1 (L1) or Operator 2 (L2)) and University of Geneva (UG) for the CBM experiments.

Upon completion of the in vitro permeation test and after a thorough cleaning of the skin surface, a 0.5 cm^2^ disc was generated from the central area of each skin sample using a punch with a diameter of 8 mm. These small skin discs were embedded in Optimal Cutting Temperature (OCT) medium, snap-frozen by immersion in isopentane chilled to its freezing point (−160 °C) in liquid nitrogen at L'Oréal. 18 skin disc samples (6 female donors, 3 samples per skin donor) were distributed to L'Oréal Operator 1 (L1, *n* = 6), L'Oréal Operator 2 (L2, n = 6), and University of Geneva (UG, n = 6). The same cryosectioning protocol was carried out at L'Oréal Laboratories by L1 or L2 using a Microm HM560 Cryostat (Thermo Scientific, Runcorn, UK) and independently at University of Geneva by UG, using a CryoStar NX70 Cryostat (Thermo Scientific, Runcorn, UK). The schematic illustration of the study design and protocol for the processing of the skin samples is presented in Fig. 1. Briefly, the skin sample was mounted in a cryotome and horizontally (XY-plane) sectioned to obtain lamellae with a thickness of 20 μm. In total, 20 lamellae were obtained from each small disc, i.e. descending from the skin surface to a depth of 400 μm in high resolution: thereby providing (theoretically) the biodistribution profile of the investigated active ingredients from the stratum corneum, epidermis and down to the upper dermis. Each lamella and the remaining skin from each sample were collected in an individual Eppendorf tube, all the tubes were stored at −20 °C before extraction at L'Oréal following the same procedure.

All samples (L'Oréal and Geneva) underwent a rigorously identical thermal history to guarantee strict comparability and eliminate any analytical bias. All tubes containing skin lamellae and extraction medium (generated at L'Oréal by L1 and L2) were immediately refrozen at −20 °C. Samples processed at University of Geneva were shipped on dry ice and stored at −20 °C upon receipt at L'Oréal prior to sample processing. The extraction and analysis were performed by the same operator at L'Oréal. Validation of extraction efficiency is presented in the Supplementary Information (Table S7). The extracts were centrifuged, filtered, and subsequently analysed by L'Oréal using the validated UHPLC-MS/MS method.

### 2.6 Data analysis

For each compound of interest, data collected from both the in vitro permeation test (permeation and total skin deposition) and the biodistribution profiles of the five active ingredients in human skin were analysed with the R software version 4.3.3 (RCoreTeam, 2023) to assess the presence of significant differences in the results obtained by the three individual operators from the two different laboratories who performed the cutaneous biodistribution experiments following an identical protocol. The detailed description of the data analysis methods is presented below in 2.6.1 and 2.6.2.

#### 2.6.1 Methods for analysing in vitro permeation test

For the data generated by each of the three operators − UG, L1, and L2 – both the amount of active ingredient permeated across the skin and the amount deposited within the skin were examined. $P_{\mathrm{kji}}$ and $D_{\mathrm{kji}}$ were denoted as the permeation and total deposition measurements for the k-th compound, j-th operator and i-th donor, respectively, which were assumed to be independent across the 6 skin donors. Wilcoxon signed-rank tests on the paired differences for each combination of operator pair and compound were performed to investigate potential discrepancies in experimental results generated by the three individual operators, which aimed to evaluate whether the median of the differences between paired observations is significantly different from zero. Namely, for a given compound $k∊\left\{1,\ldots ,5\right\}\;$and pair of operators $j,j^{\prime }∊\left\{123\right\}$ with $j\neq j^{\prime }$, the permeation and total deposition measurements were used to assess the hypotheses.

$H_{0}^{\left(P\right)}:\text{median}\left(P_{\mathrm{kji}}-P_{kj^{\prime }i}\right)=0\;$vs. $H_{0}^{\left(P\right)}:\text{median}\left(P_{\mathrm{kji}}-P_{kj^{\prime }i}\right)\neq 0,$

and $H_{0}^{\left(D\right)}:\text{median}\left(D_{\mathrm{kji}}-D_{kj^{\prime }i}\right)=0\;$vs. $H_{0}^{\left(D\right)}:\text{median}\left(D_{\mathrm{kji}}-D_{kj^{\prime }i}\right)\neq 0$*,*

where i=1,…,6, respectively. This non-parametric test is invariant to monotonic transformations of the data, such as the logarithm. Since the log-normal distribution typically provides a reasonable approximation for the permeation and total deposition data (Figs. S4-S5), paired t-tests on the log-transformed data were also performed as a parametric corroboration, assessing whether the experimental protocol yields significantly different outcomes when conducted by the three operators. The detailed results are reported in the Supplementary Information (Table S8 and Fig. S3). However, as the permeation data exhibit mild departures from normality at these small sample sizes, the Wilcoxon test remains the preferred choice for these analyses. For each set of tests (i.e. the permeation and total deposition on both the original and log scales), multiple testing through a Bonferroni multiplicity correction was employed by setting α=5%, then each test was conducted at the corrected level $\alpha_{c}=\alpha /T_{1}$, where $T_{1}=15$ denotes the total number of tests performed in each assessment (i.e. 3 operator comparisons × 5 compounds). Less stringent corrections, such as the Holm method and the Benjamini-Hochberg procedure, lead to the same conclusions, with no hypothesis rejected under any correction, and are thus omitted for brevity.

#### 2.6.2 Methods for analysing biodistribution profiles

The biodistribution profile of the five active ingredients in human skin was further analysed to obtain a more granular assessment compared to the one based on total depositions. $X_{\mathrm{kjdi}}$ were denoted as the concentration levels for the k-th compound, j-th operator, where $d∊\left\{1,\ldots ,20\right\}$ indicated the skin lamella layer and $i∊\left\{1,\ldots ,n\right\}$ represented the donor (*n* = 6). For fixed k,j,d, as customary for these types of data, the observations $X_{\mathrm{kjdi}}$ were assumed to be independent and identically distributed according to a log-normal distribution, therefore, the log-transformation of biodistribution data by setting $Y_{\mathrm{kjdi}}=\ln\left(X_{\mathrm{kjdi}}\right)∼N\left(\mu_{\mathrm{kjd}},\sigma_{\mathrm{kjd}}^{2}\right)$ for all $i∊\left\{1,\ldots ,n\right\}$ was considered. For a given compound and operator pair, rather than individually comparing each of the 20 skin lamella layers, the number of statistical tests was reduced by grouping the samples from the first 18 lamella layers, corresponding to the range of 0 to 360 μm. Therefore, for each compound k, operator j, and donor i, ${\tilde{Y}}_{\mathrm{kji}}=\frac{1}{18}\sum_{d=1}^{18}Y_{\mathrm{kjdi}}∼N\left({\tilde{\mu }}_{\mathrm{kj}},{\tilde{\sigma }}_{\mathrm{kj}}^{2}\right)$. For a given compound that the true difference in the means between two operators be $\tilde{\theta }={\tilde{\mu }}_{\mathrm{kj}}-{\tilde{\mu }}_{kj^{\prime }}$, the hypotheses.

$H_{0}:\tilde{\theta }=0\;$vs. $H_{1}:\tilde{\theta }\neq 0.$

were tested through two-sided t-tests. In this case, a nominal significance level α=5% was considered and each test with an adjusted level at $\alpha_{c}=\alpha /T_{1}$ based on the Bonferroni correction was performed, where $T_{1}=15$ accounting for the total number of tests (i.e. 3 operator comparisons × 5 compounds).

Besides, for each operator pair, the spatial distributions of the active ingredients were grouped into three main anatomical regions: epidermis (ED; 0–80 μm), dermo-epidermal junction (DEJ; 100–200μm), and dermis (DM; 220–360μm) for a more detailed assessment of intra- and inter-laboratory comparisons; Dermal-Epidermal Junctions (DEJ) joins the epidermis to the dermis and contains the cutaneous basement membrane, which is an inter-ridge dimension with a depth of 100–200 μm roughly (Aleemardani et al., 2021), therefore, the regions were categorized as presented. Specifically, for each compound k and operator j, partial averages across donors were calculated to construct ${\bar{Y}}_{\mathrm{kjg}}=\frac{1}{\left|R_{g}\right|}\sum_{d∊R_{g}}Y_{\mathrm{kjdi}}∼N\left({\bar{\mu }}_{\mathrm{kjg}},{\bar{\sigma }}_{\mathrm{kjg}}^{2}\right)$, where $R_{g}\;$for g∊{1, …, 3} represents the respective set of skin lamella layers for one of the regions of interest – ED, DEJ or DM, and $|R_{g}|$ denotes the cardinality of each set (4, 6 and 8, respectively). For a given compound k and operators' comparison j vs. $j^{’}$, let ${\bar{\theta }}_{g}={\bar{\mu }}_{\mathrm{kjg}}-{\bar{\mu }}_{\mathrm{kj}\prime g}$ indicate the true difference in means for the g-th region. Then the hypotheses

$H_{0}^{\left(g\right)}:{\bar{\theta }}_{g}=0$ vs. $H_{1}^{\left(g\right)}:{\bar{\theta }}_{g}\neq 0$,

which have been evaluated by using two-sided t-tests at a nominal significance level of α=5%, were used to test each skin region g∊{1, …, 3}. To account for multiple testing, the Bonferroni correction, using an adjusted significance level of $\alpha_{c}=\alpha /T_{2}$, where $T_{2}=45$ (i.e. 3 anatomical regions × 3 operator comparisons × 5 compounds) was applied.

Also in this case, less stringent corrections, such as the Holm method and the Benjamini-Hochberg procedure, lead to the same conclusions for both sets of tests (i.e., $T_{1}=15$ and $T_{2}=45$), with no hypothesis rejected, and they are thus omitted for brevity.

## 3 Results and discussion

### 3.1 Analytical methods

The analytical methods for the quantification of proxylane, minoxidil, oxybenzone, caffeine and compound X were shown to be specific, sensitive, linear, accurate, and precise over a broad concentration range (∼1–2000 ng/mL) for the skin permeation samples and biodistribution samples. The exact upper and lower limits of quantification (ULOQ and LLOQ) for each of the five investigated active ingredients with respect to the permeation and biodistribution samples are shown in Table 4. The complete results and details are presented in the Supplementary Information (Table S3).

**Table 4:** Upper and lower limits of quantification (ULOQ and LLOQ) of the five investigated active ingredients.

Limits of quantification (ng/mL)	Proxylane	Caffeine	Minoxidil	Compound X	Oxybenzone
ULOQ	2000	2000	2000	2000	2000
LLOQ (permeation samples)	4.35	0.518	0.581	2.07	0.615
LLOQ (biodistribution samples)	0.547	5.16	1.04	0.488	0.996

### 3.2 Evaluation of in vitro skin permeation and total deposition

The amounts of the five active ingredients permeated across the skin (cutaneous permeation) and deposited in the skin (total deposition) were quantified using the validated analytical method, and the results from the samples generated by the three operators (UG, L1, and L2) are presented in Table 5.

**Table 5:** Permeation and total deposition (mean ± SD, *n* = 6) of the five active ingredients (proxylane, caffeine, minoxidil, compound X, and oxybenzone) generated by the three operators (UG, L1, and L2). Data were collected from paired samples.

Measurement	Compound	UG	L1	L2
Permeation (μg/cm^2^)	Proxylane	0.06 ± 0.01	0.07 ± 0.01	0.09 ± 0.06
	Caffeine	4.00 ± 1.39	4.01 ± 1.50	3.46 ± 2.04
	Minoxidil	0.15 ± 0.23	0.22 ± 0.42	0.22 ± 0.41
	Compound X	0.45 ± 0.37	0.50 ± 0.59	0.51 ± 0.66
	Oxybenzone	2.29 ± 1.11	2.67 ± 1.83	2.59 ± 2.02
Deposition (μg/cm^2^)	Proxylane	0.23 ± 0.17	0.53 ± 0.31	0.33 ± 0.12
	Caffeine	0.52 ± 0.18	0.60 ± 0.20	0.32 ± 0.10
	Minoxidil	0.27 ± 0.25	0.41 ± 0.34	0.30 ± 0.12
	Compound X	0.77 ± 0.48	0.90 ± 0.63	0.84 ± 0.19
	Oxybenzone	1.73 ± 0.45	2.08 ± 0.51	2.02 ± 0.32

**Fig. 2:**
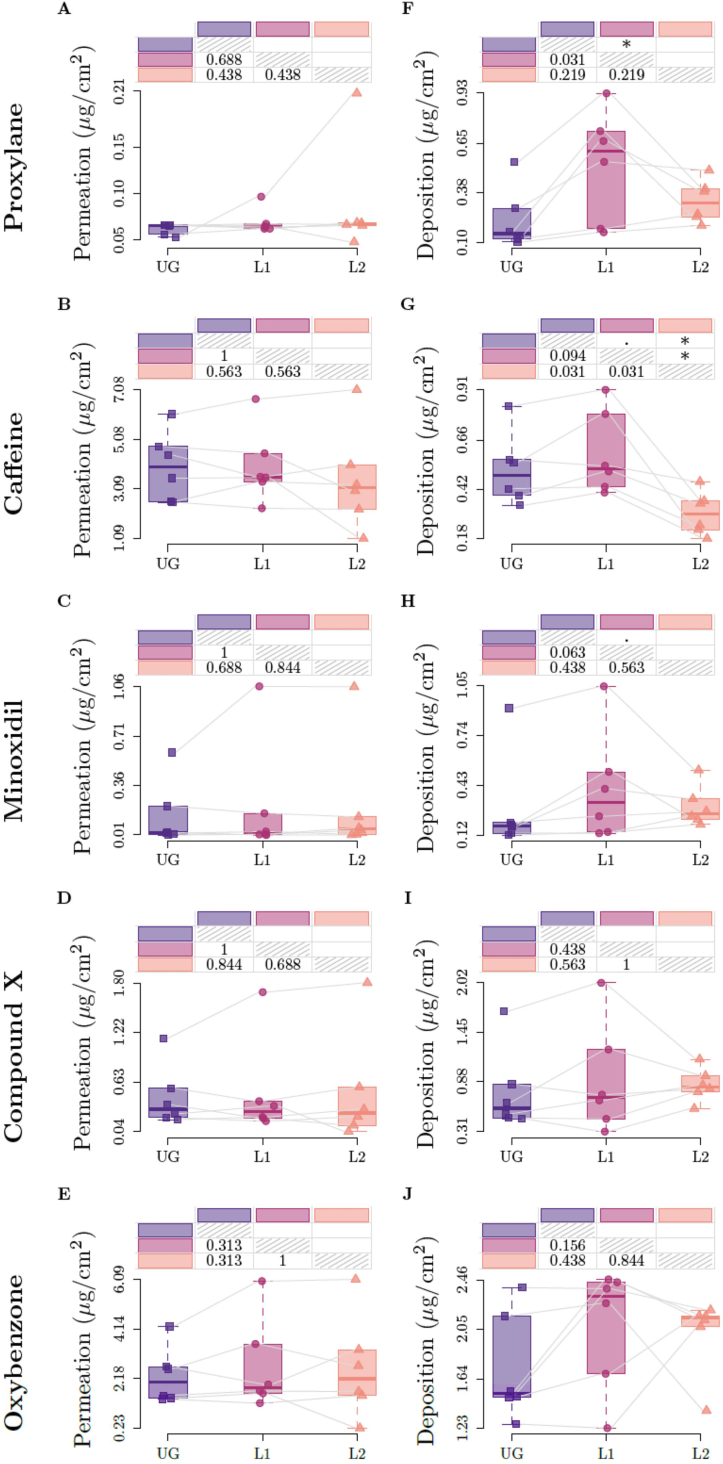
Paired skin permeation (A-E) and total deposition (F-J) data for proxylane (A, F), caffeine (B, G), minoxidil (C, H), compound X (D, I), oxybenzone (E, J) generated by University of Geneva (UG), L'Oréal Operator 1 (L1), and L'Oréal Operator 2 (L2) for intra and inter-laboratory validation (mean ± SD, *n* = 6).

For each operator and each active ingredient of interest, the permeation and total deposition paired data are presented in Fig. 2, with individual data points horizontally jittered and grey lines connecting paired human skin donors to highlight their relationships for a clearer visualization; *p*-values associated to the Wilcoxon signed-rank tests between operator pairs (UG vs. L1, UG vs. L2, and L1 vs. L2) and compounds, also their significance (using the conventional notation: “ . ”, “ * ”, “ ** ” and “ *** ” for 0.05 < *p* ≤ 0.1, 0.01 < *p* ≤ 0.05, 0.001 < *p* ≤ 0.01, and *p* ≤ 0.001respectively) were introduced to test the hypotheses $H_{0}^{\left(P\right)}$ and $H_{0}^{\left(D\right)}\;$as presented in Section 2.6.1. All *p*-values are larger than the corrected level $\alpha_{c}=0.05/15\approx 0.003$, therefore, there were no statistically significant differences present in either the cutaneous permeation or total deposition measurements of the five compounds generated by the three individual operators.

For each pairwise comparison across the three individual operators, *p*-values obtained from paired, two-sided Wilcoxon signed-rank tests are reported, and tests are performed at the corrected significance level$\;\alpha_{c}=0.05/15\approx 0.003$ ($\alpha_{c}=\alpha /T_{1}$, where $T_{1}=15$ accounting for 3 operator comparisons × 5 compounds). The symbols “.”, “*”, “**” and “***” for 0.05 < *p* ≤ 0.1, 0.01 < *p* ≤ 0.05, 0.001 < *p* ≤ 0.01, and *p* ≤ 0.001tiered *p*-values smaller than 0.1, 0.05, 0.01 and 0.001, respectively. Interestingly, total depositions for proxylane (*p* = 0.031 comparing UG vs. L1, Fig. 2.F) and caffeine (*p* = 0.031 comparing L2 vs. UG, and *p* = 0.031 comparing or L2 vs. L1, Fig. 2.G) report the smallest *p*-values, which however remain larger than the corrected significance level $\alpha_{c}$ (0.003). The variabilities in the total skin deposition may be related to the washing procedure used to remove all chemical remaining on the skin surface, which has a more pronounced ability to extract water soluble chemicals such as proxylane and caffeine from the skin (Wargniez et al., 2022). Overall, the absence of difference in the permeation samples (all generated and analysed at L'Oréal by the same operator) confirmed that IVPT test was conducted in a reproducible manner among the three experimental groups, which laid the foundation for the further comparison of CBM; the lack of significant difference in the total deposition data obtained following the identical experimental protocols suggested the reproducibility of CBM at both intra-laboratory level (i.e. between L'Oréal Operators, L1 vs. L2) and inter-laboratory level (i.e. between L'Oréal Operators and University of Geneva, L1 vs. UG and L2 vs. UG). Furthermore, these results align with those obtained from the t-tests on log-transformed data are presented in the Supplementary Information (Fig. S3).

### 3.3 Biodistribution profile

**Fig. 3:**
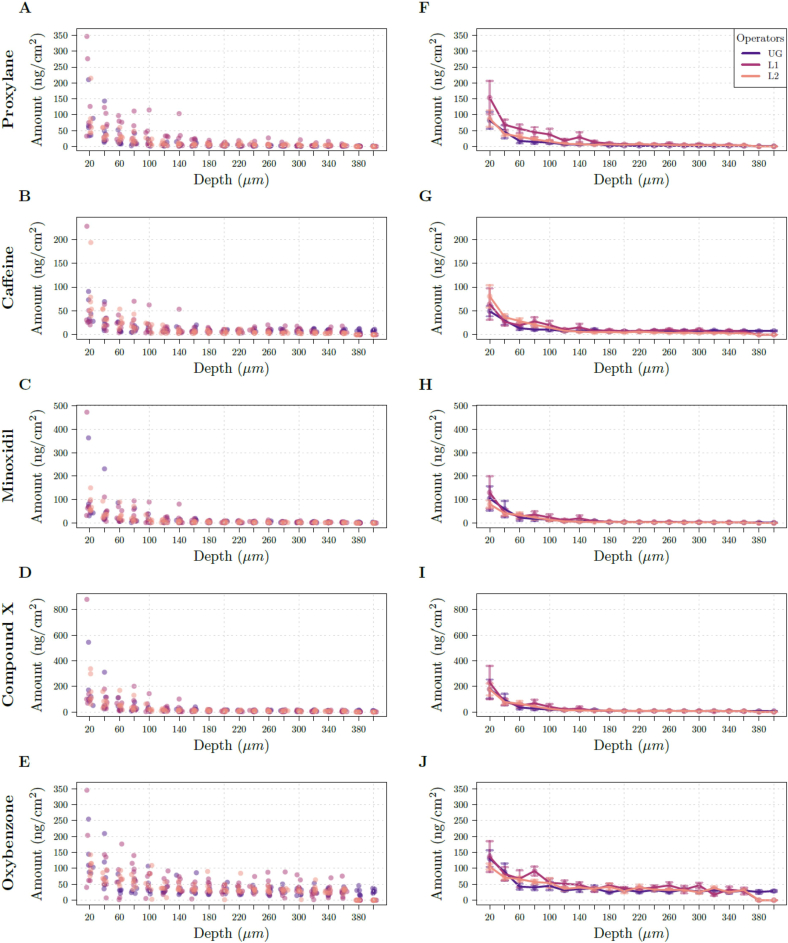
Individual biodistribution profile of A) proxylane, B) caffeine, C) minoxidil, D) compound X, E) oxybenzone (and the corresponding mean profiles, panels F-J) obtained by University of Geneva (UG; *n* = 6, mean ± SD), L'Oréal Operator 1 (L1; n = 6, mean ± SD), and L'Oréal Operator 2 (L2; n = 6, mean ± SD) for intra-laboratory and inter-laboratory validation.

To further understand the spatial distribution of the topically applied active ingredients in the skin, the CBM was used to generate profiles of the amount of each investigated active ingredient as a function of position down to a depth of ∼400 μm (20 lamellae each with a thickness of 20 μm), encompassing the stratum corneum, viable epidermis and upper dermis. Fig. 3 presents the amounts of the five investigated ingredients − proxylane, caffeine, minoxidil, compound X, oxybenzone – as a function of skin depth after application for 16 h. Visual inspection of the profiles served as a useful check on the quality of the results obtained – for example, to detect very clear outliers in the data. Generally, the biodistribution profiles of all the investigated ingredients were similar between UG, L1, and L2. Taking UG as a reference, based on a qualitative assessment, better reproducibility was achieved by L2 as most of the data points in the two groups were superimposed and were clustered around the mean value; however, L1 displayed a higher dispersity in the values of all the investigated ingredients and mild outliers could be noticed in almost each data set, despite the fact that samples from L1 and L2 were manipulated following the same protocol using an identical cryosection machine in the same laboratory. It was possible that instrument-specific variability might have resulted from the use of two different cryostat models — Microm HM560 and CryoStar NX70; however, the lack of any difference points to the robustness of the method.

**Fig. 4:**
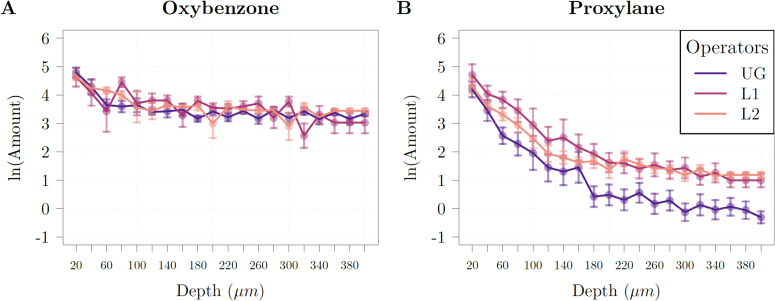
Biodistribution profile of A) oxybenzone and B) proxylane obtained by University of Geneva (UG; n = 6, mean ± SD), L'Oréal Operator 1 (L1; n = 6, mean ± SD), and L'Oréal Operator 2 (L2; n = 6, mean ± SD) for intra-laboratory and inter-laboratory validation. Data are presented after transformation of the amount per unit area (reported in Fig. 3) to a natural logarithm scale.

Interestingly, when setting the y-axis to a natural log scale, it is clear that the biodistribution profile of the most lipophilic compound – oxybenzone (log_10_P = 4.03) was comparatively constant with fluctuations in a small range (Fig. 4.A), while the amounts of the most hydrophilic molecule − proxylane (log_10_P = −1.51), were dramatically decreasing as a function of skin depth (Fig. 4.B). These results confirmed that the barrier function of stratum corneum was the limiting step in regulating cutaneous penetration of proxylane, only a small fraction of the hydrophilic compound could penetrate through the outermost layer; however, the more lipophilic oxybenzone could diffuse more freely through the extracellular lipid matrix, enabling greater amounts to reach the epidermis and upper dermis.

### 3.4 Statistical model for the analysis of the biodistribution profiles

**Fig. 5:**
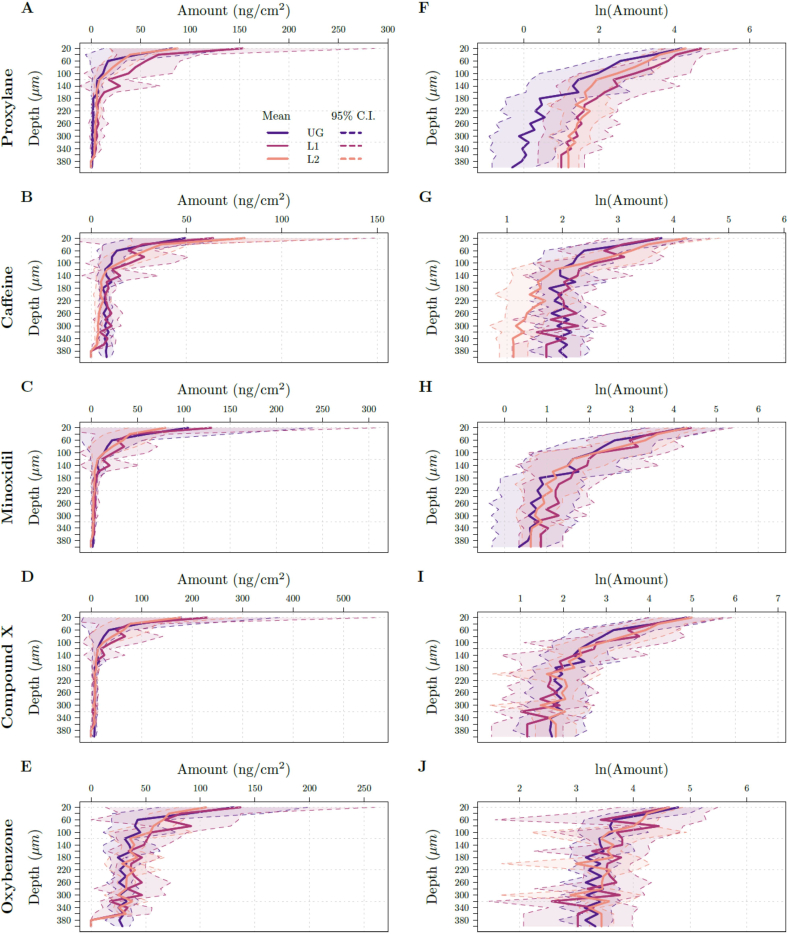
Biodistribution profile presented on the original scale (A-E), and after a logarithmic transformation (F-J) for proxylane (A, F), caffeine (B, G), minoxidil (C, H), compound X (D, I), oxybenzone (E, J). Data were generated by the three operators — University of Geneva (UG), L'Oréal Operator 1 (L1), and L'Oréal Operator 2 (L2) for intra- and inter-laboratory validation. The solid lines indicate the mean value of the amount of drug in each layer as determined by each operator and the dashed lines represent the 95% point-wise confidence intervals for the means.

**Fig. 6:**
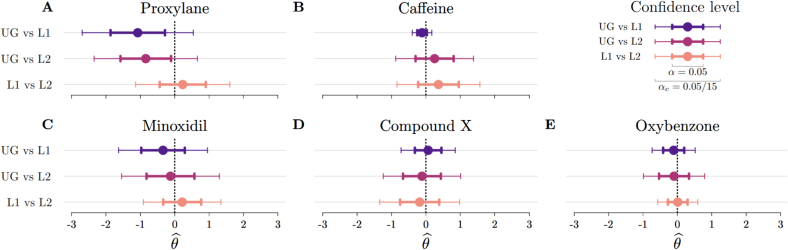
Confidence intervals for log-transformed biodistribution profiles (shown in Fig. 4) of A) proxylane*,* B) caffeine, C) minoxidil, D) compound X, and E) oxybenzone. Data were generated by the three operators — University of Geneva (UG), L'Oréal Operator 1 (L1), and L'Oréal Operator 2 (L2).

**Fig. 7:**
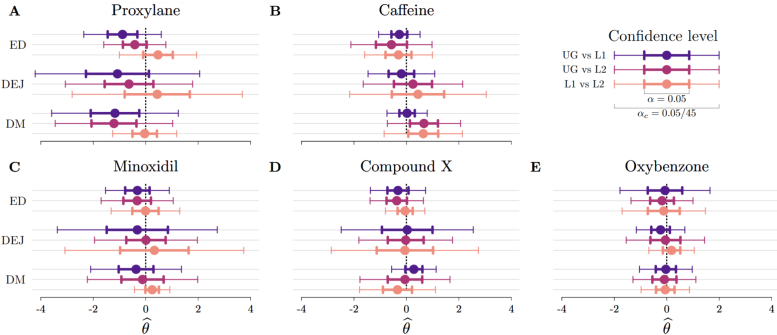
Confidence intervals for log-transformed biodistribution profiles of A) proxylane, B) caffeine, C) minoxidil, D) compound X, and E) oxybenzone. Data were generated by the three operators — University of Geneva (UG), L'Oréal Operator 1 (L1), and L'Oréal Operator 2 (L2) across skin regions. Skin depths are grouped in terms of epidermis (ED; 0–80 μm), dermo-epidermal junction (DEJ; 100–200 μm), and dermis (DM; 220–360 μm).

A statistical analysis was performed to further validate the reproducibility of the experimental protocol at both intra- and inter-laboratory levels. The biodistribution profiles of each compound of interest generated by the three operators are presented on the original scale (Fig. 5. A-E) and after a natural logarithmic transformation (Fig. 5. F-J), respectively. The solid lines indicate the mean value of the amount of drug (x-axis) in each layer (y-axis) as determined by each operator, and the corresponding dashed lines represent the (asymptotic) 95% point-wise confidence intervals for the means. On the original scale, the biodistribution profiles exhibit a characteristic exponential decay from the outmost skin layers, resulting in a highly right-skewed distribution of the data, combined with a decreasing variability at deeper skin layers. For these types of data, the log-normal distribution typically offers a reasonable approximation (Keene, 1995). Indeed, on the natural logarithmic scale, the transformed measurements are characterized by more homogeneous variances, and the normal distribution provides a suitable approximation across skin depths (Fig. S6-S7). This data transformation is useful for the valid application of subsequent parametric tests of hypotheses that rely on a normal assumption, such as the *t*-tests provided in Fig. 6, Fig. 7, which serve the purpose of quantitatively comparing compound delivery between different operators.

A statistical analysis was conducted to assess the set of hypotheses $H_{0}\;\mathrm{vs}.H_{1}$ (Section 2.6.2) across operator pairs for each compound; the confidence intervals for the difference in log-concentration in each skin lamella from depths ranging from 20 to 360 μm are presented in Fig. 6, all confidence intervals at the corrected significance level $\alpha_{c}\approx 0.003$ cover the value of zero, therefore, none of those null hypothesis could be rejected. This result further confirmed the analyses on permeation and total deposition data presented in Section 3.2.

Furthermore, the set of hypotheses$\;H_{0}^{\left(g\right)}\;\mathrm{vs}.H_{1}^{\left(g\right)}$ (Section 2.6.2) for each compound and laboratory pair was assessed. Fig. 7 reports confidence intervals for the difference in log-concentrations across ED, DEJ and DM regions. Also in this case, all confidence intervals at the corrected level $\alpha_{c}=\frac{0.05}{45}\approx 0.001\;$include the value of zero, indicating that none of the null hypotheses can be rejected. Therefore, no statistically significant differences associated with the experimental protocols across the data generated by the three operators were detected.

## 4 Conclusion

We have previously used the CBM to evaluate and to optimize novel formulations and to benchmark them against approved products (Lapteva et al., 2014; Chen et al., 2016; Lapteva et al., 2019; Kandekar et al., 2019; Quartier et al., 2021; Dahmana et al., 2021; Gou et al., 2022). It has also been proposed as a more physiologically/pharmacologically relevant in vitro approach to establish bioequivalence than the IVPT tests since it provides data that compare the amounts delivered in the skin and as a function of position in the epidermis and dermis (Quartier et al., 2019), which are the target compartments for topically applied dermatological drugs (and for skin care products). As a first step, for both applications, it was deemed essential to demonstrate the consistency and reproducibility of intra- and inter-laboratory results – hence, the motivation for this collaborative study. The data generated using a cassette-based formulation containing five chemically distinct active molecules with different physicochemical properties and a > 350,000-fold difference in lipophilicity and applied under finite dose conditions to human skin indeed confirmed that skilled operators could produce consistent cutaneous biodistribution results with no statistically significant differences. This opens the door to more widespread use of this method which can provide detailed quantitative information with respect to the spatial distribution of active molecules (and excipients) in the epidermis and dermis and could facilitate topical formulation development and provide insight with respect to differences in efficacy observed in vivo.

## CRediT authorship contribution statement

**Si Gou:** Writing – original draft, Validation, Methodology, Investigation, Data curation. **Anne Moustie:** Writing – original draft, Validation, Methodology, Investigation, Data curation. **Kevin Caché:** Writing – original draft, Methodology, Investigation, Data curation. **Luca Insolia:** Writing – original draft, Formal analysis. **Jun Wu:** Writing – original draft, Formal analysis. **Sébastien Grégoire:** Writing – review & editing, Resources, Methodology, Conceptualization. **Stéphane Guerrier:** Writing – original draft, Formal analysis. **Yogeshvar N. Kalia:** Writing – review & editing, Supervision, Resources, Project administration, Conceptualization.

## Declaration of competing interest

The authors declare the following financial interests/personal relationships which may be considered as potential competing interests:

Anne Moustie, Kevin Caché, and Sébastien Grégoire, are/were employees of L'Oréal Research and Innovation at the time of writing.

## Data Availability

Data will be made available on request.
