# Supplementary material for: Cutaneous biodistribution of topically applied active ingredients: Evaluation of intra- and inter-laboratory reproducibility
Source: Int J Pharm X. 2026 Jul 16;12:100618. doi: 10.1016/j.ijpx.2026.100618 (PMC13396900; doi:10.1016/j.ijpx.2026.100618)
Supplement: Supplementary file 1 — Supplementary material for this article can be found online at https://doi.org/10.1016/j.ijpx.2026.100618. [file mmc1.docx]

**SUPPLEMENTARY INFORMATION**

**Cutaneous biodistribution of topically applied active ingredients: Evaluation of intra- and inter-laboratory reproducibility**

**Si Gou^1,2^, Anne Moustie^3^, Kevin Caché^3^, Luca Insolia^1,2,4^, Jun Wu^5^, Sébastien Grégoire^3^, Stéphane Guerrier^1,2,4^, Yogeshvar N. Kalia^1,2 *^**

^1^ School of Pharmaceutical Sciences, University of Geneva, 1211 Geneva, Switzerland.

^2^ Institute of Pharmaceutical Sciences of Western Switzerland, University of Geneva, 1211 Geneva, Switzerland.

^3^ L’Oréal R&I France, 1 Av. Eugène Schueller, 93600 Aulnay-Sous-Bois, France.

^4^ Department of Earth Sciences, University of Geneva, 1206 Geneva, Switzerland.

^5^ Geneva School of Economics and Management, University of Geneva, 1211 Geneva, Switzerland

*Corresponding Author

Prof. Yogeshvar N. Kalia

School of Pharmaceutical Sciences

University of Geneva

1 Rue Michel-Servet,

1211 Geneva, Switzerland

Email: [yogi.kalia@unige.ch](mailto:yogi.kalia@unige.ch)

1. **Validation of UHPLC-MS/MS method**

The validation of the analytical method was performed as per *Bioanalytical FDA guidelines*: the selectivity and specificity, sensitivity, precision and accuracy were tested.

- 1. **Selectivity and specificity**
     1. Selectivity

Five different ion transitions were followed in MRM (Multiple reaction monitoring) mode for the detection of oxybenzone, minoxidil™, caffeine, compound X, and proxylane™, respectively. **Figure SI1** shows the chromatogram obtained after injecting a mix of the five compounds in methanol at 2000 ng/mL. As expected, the five copounds eluted at different retention time, and there is only one peak in each MRM trace, meaning that the MS/MS detection was selective for each compound.


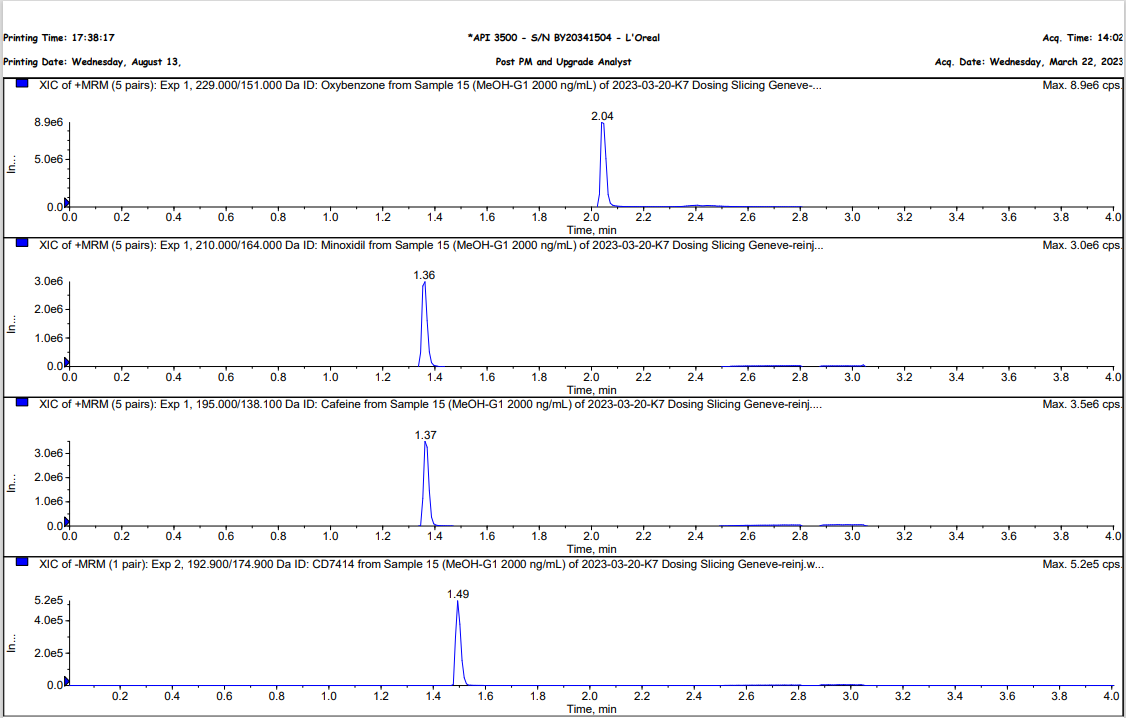


**D**

**C**

**B**

**A**


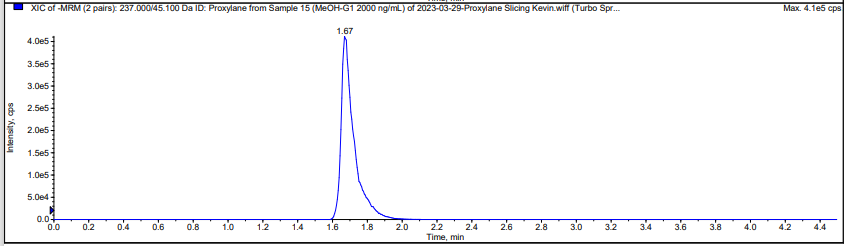
**Figure S1.** Chromatogram of **A)** Oxybenzone, **B)** Minoxidil, **C)** Caffeine, **D)** Compound X, and **E)** Proxylane at 2000ng/mL in methanol

**E**

- - 1. Specificity

Specificity is defined as “the absence of interference at the signal of the active in the blank matrix of a biological sample.” In this study, specificity is evaluated by comparing the signal in the blank matrix with the signal at the LLOQ in the biological matrix. According to *Bioanalytical FDA guidelines*, the specificity is validated only if the ratio of peak area in the blank matrix comparing to the peak area at the LLOQ is less than 20% ― the response in the blank is considered as negligible. **Table S1** presented the specificity evalution of the five compounds of interest in the presence of biological matrix extracted from skin epidermis and dermis. There were no inteference from the skin matrix for oxybenzone, minoxidil, compound X, proxylane, however, endogenous caffeine was detected at significant levels both in epidermis and dermis. Therefore, the LLOQ of caffeine was corrected to fullfill the acceptability criteria of 20%.

**Table S1**. Specificity evaluation of the five compounds of interest

| **Compounds** |  | **Specificity** | |
| --- | --- | --- | --- |
|  |  | Epidermis | Dermis |
| Oxybenzone |  | no peak | no peak |
| Minoxidil |  | no peak | no peak |
| Caffeine |  | peak in blank matrix > 20% peak area at LLOQ: endogenous | |
| Compound X |  | no peak | no peak |
| Proxylane |  | no peak | no peak |

The matrix effect in LC-MS/MS describes the influence of the chemical environment of a charged ion on the intensity of its signal. This can result in either suppression or enhancement of the signal depending on the nature of the sample. In this study, the matrix effect is evaluated by comparing the signal in a blank matrix boosted at a defined concentration to the signal in the solvent, used for the calibration range, boosted at the same defined concentration. In accordance with FDA guidelines for bioanalytical assays, the matrix effect is validated only if the ratio of the peak area in the boosted blank matrix to the peak area in the solvent is less than 15%. The effects of the skin matrix on ion suppression or ion enhancement were also determined, which were considered as negligeable given that they are inferior to ± 15% for each compound of interest as presented in **Table S2**.

**Table S2**. Matrix effects evaluation

| **Compounds** |  | **Matrix effect %** | |
| --- | --- | --- | --- |
|  |  | Epidermis | Dermis |
| Oxybenzone |  | 5.50 ± 3.81 | 0.46 ± 2.63 |
| Minoxidil |  | 7.09 ± 4.05 | -7.93 ± 3.66 |
| Caffeine |  | 2.61 ± 3.66 | 0.89 ± 8.31 |
| Compound X |  | -2.84 ± 4.98 | -3.69 ± 5.56 |
| Proxylane |  | 1.70 ± 8.31 | -11.9 ± 2.97 |

**1.2 Calibration curve**

Calibration curves for proxylane, minoxidil, oxybenzone, caffeine and compound X were constructed over the range around 0.5-2000 ng/mL, standards were prepared in MeOH and sodium chloride buffered saline (0.9 %, w/v) containing 0.25% Tween 80 (receiver medium), for the quantification of skin extraction and permeation samples, respectively. The peak area ratio (when internal standard is available) or area (cps) was plotted against the respective concentrations (ng/mL), data indicated in **Figure S2.**


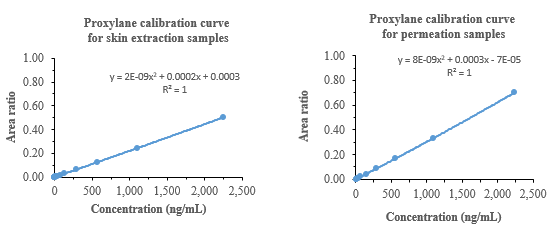

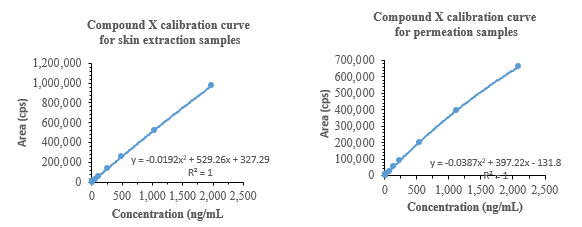

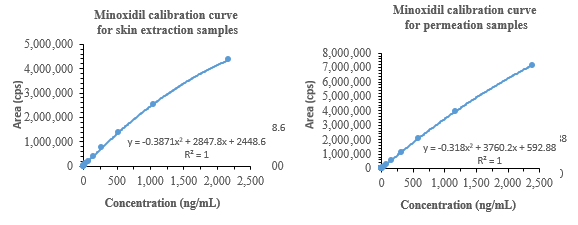

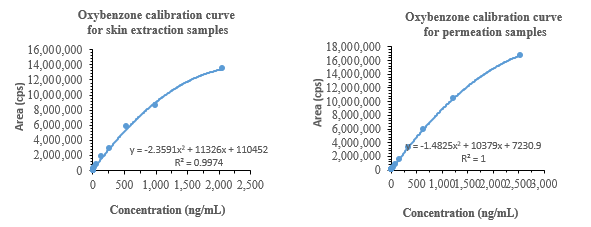

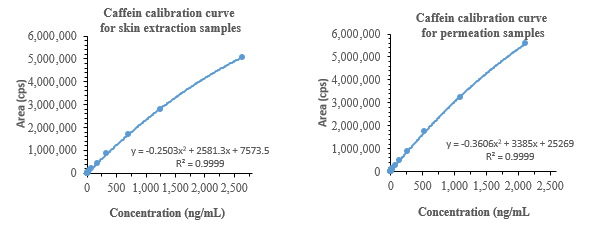


**Figure S2.** Calibration curves – Peak area ratio or area (cps) as a function of concentration (ng/mL) for proxylane, minoxidil, oxybenzone, caffeine and compound X (concentration range ~1-2000 ng/mL). The exact upper and lower limits of quantification (ULOQ and LLOQ) for each of the five compounds with respect to the permeation and skin extraction samples are shown in **Table S3**.

**1.3 Limit of detection and limit of quantification**

The limit of detection (LOD) and limit of quantification (LOQ) were determined using the quadratic regression method and the data are presented in **Table S3**. For each compound, there are 2 calibration curves (as indicated in section 1.2) one to quantify skin extraction and the other to quantify permeation samples. It should be noticed that the LLOQ of caffeine was re-evaluated based on endogenous concentrations in the epidermis and dermis, which were increased from 0.645 ng/mL to 5.16 ng/mL.

**Table S3.** LOD and LOQ for the five compounds of interest

| **Compounds** |  | **LOD (ng/ml)** | |  | **LOQ (ng/ml)** | |
| --- | --- | --- | --- | --- | --- | --- |
|  |  | Permeation samples | Skin extraction samples |  | Permeation samples | Skin extraction samples |
| Oxybenzone |  | 0.615 | 0.996 |  | 0.615 | 0.996 |
| Minoxidil |  | 0.581 | 1.04 |  | 0.581 | 1.04 |
| Caffeine |  | 0.518 | 5.16 |  | 0.518 | 5.16 |
| Compound X |  | 2.07 | 0.488 |  | 2.07 | 0.488 |
| Proxylane |  | 4.35 | 0.547 |  | 4.35 | 0.547 |

**1.4 Precision and accuracy**

The precision of the analytical method describes the closeness of repeated individual measures of analyte. It is expressed as the coefficient of variation (RSD). The accuracy of a method determines the difference between the analytical value and the theoretical value. Accuracy is determined by comparing a theoretical concentration value with the experimental value. It is expressed as a percentage of recovery. Precision and accuracy are evaluated on 9 preparations (3 preparations at 3 levels) covering the validation range. For the method to be considered as accurate, the mean concentration should be within 15% of the nominal values, except for the LLOQ which should be within 20% of the nominal value. For the method to be considered as precise, RSD value should not exceed 15%, except for the LLOQ which should not exceed 20%. The results are presented in tables **Table** **S4** for sample in skin extraction samples solvent and in **Table S5** for permeation samples solvent**.**

**Table S4.** Precision and accuracy of the analytical method for the five compounds of interest in skin extraction samples solvent (Mean ± SD, n=3).

|  | Theoretical  (ng/mL) | Measured (ng/mL) | Accuracy % (measured/nominal) | Precision on measured (RSD %) |
| --- | --- | --- | --- | --- |
| Caffeine | 10.3 | 10.1 ± 0.50 | 1.5 | 4.89 |
|  | 165 | 169 ± 8.50 | -2.4 | 5.06 |
|  | 660 | 662 ± 30.1 | -0.3 | 4.54 |
| Oxybenzone | 1.99 | 1.90 ± 0.26 | 5.7 | 14.0 |
|  | 128 | 132 ± 1.20 | -2.9 | 0.88 |
|  | 510 | 522 ± 7.60 | -2.4 | 1.46 |
| Minoxidil | 4.18 | 3.80 ± 0.20 | 9.6 | 5.19 |
|  | 134 | 136 ± 7.40 | -1.7 | 5.41 |
|  | 535 | 526 ± 17.5 | 1.7 | 3.33 |
| Compound X | 3.91 | 4.00 ± 0.42 | -2.1 | 10.7 |
|  | 125 | 122 ± 11.0 | 2.1 | 9.00 |
|  | 500 | 483 ± 24.8 | 3.5 | 5.15 |
| Proxylane | 4.38 | 4.20 ± 0.59 | 4.0 | 14.0 |
|  | 280 | 281 ± 5.20 | -0.4 | 1.85 |
|  | 560 | 560 ± 13.1 | 0.0 | 2.34 |

**Table S5.** Precision and accuracy of the analytical method for the five compounds of interest in permeation samples solvent (Mean ± SD, n=3)

|  | Theoretical  (ng/mL) | Measured (ng/mL) | Accuracy % (measured/nominal) | Precision on measured (RSD %) |
| --- | --- | --- | --- | --- |
| Caffeine | 15.5 | 15.7 ± 2.10 | -1.1 | 13.4 |
|  | 125 | 133 ± 7.20 | -6.4 | 5.42 |
|  | 500 | 497 ± 31.4 | 0.7 | 6.32 |
| Oxybenzone | 7.80 | 8.40 ± 1.08 | -7.6 | 12.9 |
|  | 125 | 139 ± 16.3 | -10.9 | 11.7 |
|  | 500 | 539 ± 77.1 | -7.8 | 14.3 |
| Minoxidil | 7.80 | 8.70 ± 0.83 | -12.0 | 9.53 |
|  | 125 | 136 ± 14.5 | -9.1 | 10.6 |
|  | 500 | 512 ± 59.4 | -2.5 | 11.6 |
| Compound X | 7.80 | 8.40 ± 0.28 | -7.3 | 3.38 |
|  | 125 | 122 ± 16.0 | 2.7 | 13.2 |
|  | 500 | 508 ± 24.2 | -1.7 | 4.76 |
| Proxylane | 7.81 | 6.80 ± 0.51 | 12.7 | 7.54 |
|  | 132 | 139 ± 7.00 | -5.1 | 5.07 |
|  | 481 | 503 ± 13.1 | -4.6 | 2.59 |

1. **Solubility test**

The receptor fluid represents deeper cutaneous layers and systemic circulation, which should not limit the compound diffusion, not interfere with analytical method, and maintain skin integrity. In order to ensure appropriate sink conditions, the solubility of the five active ingredients investigated in 0.9 % NaCl + 0.25% Tween80 was determined. Approximately 2000 mg of proxylane was dispersed in 10 mL of the medium, 200 mg of caffeine was dispersed in 10 mL of the medium, while 10 mg of minoxidil, compound X, and oxybenzone were pooled in 10 mL of the medium and left under stirring at 32°C for 16 h in triplicate. Saturation was observed for caffeine, minoxidil, compound X, and oxybenzone) but not in the case of proxylane. The excess of powder was removed by filtration on Millex HV 0.45µm filter, and the five compounds were quantified using the validated UHPLC-MS/MS method. The solubility results are shown in **Table S6**.

**Table S6.** Solubility for the five compounds of interest (Mean ± SD, n=3)

| **Compounds** | **Solubility (mg/mL)** | |
| --- | --- | --- |
| Proxylane | | ≥ 80.1 ± 3.17 |
| Caffeine | | 19.6 ± 6.35 |
| Minoxidil | | 0.81 ± 0.02 |
| Compound X | | 0.99 ± 0.25 |
| Oxybenzone | | - 1. ± 0.04 |

1. **Validation of the extraction procedure**

During the experiments, 20 lamellae (20 µm thickness) and a remaining skin (400 µm thickness) were obtained. Since it was not possible to evaluate extraction efficiency from a single lamella, we used the recoveries determined previously for conventional in vitro permeation studies. These were also used for the remaining 400 µm thick dermis samples.

The validation of extraction efficiency consists in comparing a skin sample (epidermis and dermis) boosted with a known volume of active ingredient and then extracted, with a skin sample extracted and then boosted with the same known volume of active ingredient. This comparison is made at two concentrations: a low concentration equivalent to 5 to 10 times the LLOQ and a high concentration equivalent to a mid-range. If the extraction recovery at the two concentrations tested are closed, a mean recovery is calculated. This recovery must be greater than or equal to 50% with a CV less than 15%.

For the five compounds the concentrations tested were 61.7 ng/mL and 185ng/mL in epidermis and 11.1ng/mL and 104.2 ng/mL in dermis. The mean extraction recovery in the different compartments is indicated in **Table S7**.

**Table S7**. Extraction recovery efficiency (Mean ± SD, n=6)

| **Compound** | Epidermis | Dermis |
| --- | --- | --- |
| Proxylane* | 90.3 ± 7.91 | 88.5 ± 3.39 |
| Caffeine | 100 ± 7.03 | 93.7 ± 10.23 |
| Minoxidil | 96.9 ± 2.72 | 82.7 ± 3.13 |
| Compound X | 86.5 ± 14.8 | 51.2 ± 14.4 |
| Oxybenzone | 97.2 ± 3.29 | 89.0 ± 6.18 |

**Concentration tested for Epidermis-Dermis : 6.35 and 324 ng/mL*

1. **Evaluation of log-transformed permeation and total deposition data**

As customary for these types of data, we assume that they are independent across donors and follow a log-normal distribution. Therefore, after a log-transformation, the data are normal, in the sense that $ln(P_{kj1}), ..., ln(P_{kjn})\sim N(\mu_{P,kj}, \sigma_{P,kj}^{2})$ and$ln(D_{kj1}), ..., ln(D_{kjn})\sim N(\mu_{D,kj}, \sigma_{D,kj}^{2})$. The boxplots on paired data in **Figure S2** illustrate that this appears to be a reasonable approximation for the transformed data. Then, similarly to **Section 2.6.1**, for each compound $k∊\{1, \ldots, 5\}$and pair of labs $j,j^{'}∊\{1,2,3\}$ with $j\neq j^{'}$, we let $\theta_{P}\equiv\mu_{P,kj}-\mu_{P,kj^{'}}$ and $\theta_{D}\equiv\mu_{D,kj}-\mu_{D,kj^{'}}$ denote the true unknown difference in population means for permeation and total depositions, respectively. Thus, we considered the hypotheses $H_{0}^{(P^{*})}: \theta_{P}=0$vs. $H_{0}^{(P^{*})}: \theta_{P}\neq0$, and $H_{0}^{(D^{*})}: \theta_{D}=0$vs. $H_{0}^{(D^{*})}: \theta_{D}\neq0$, and performed two-sided $t$-tests to assess each set of hypotheses. For each of these, we accounted for multiple testing through a Bonferroni multiplicity correction. In particular, we set $\alpha=5\%$ and then performed each test at the corrected level $\alpha_{c}=\alpha/T_{1}$, where $T_{1}=15$ denotes the total number of tests performed in each assessment (i.e., $3 lab comparisons\times5 \mathrm{compounds}$).

**Table S8**. The logarithmic transformation of the cutaneous permeation and total deposition (Mean ± SD, n = 6) of the five active ingredients (proxylane, caffeine, minoxidil, compound X, and oxybenzone) generated by the three operators (UG, L1, and L2). Data were collected from paired samples.

| **Measurement** | **Compound** | **UG** | **L1** | **L2** |
| --- | --- | --- | --- | --- |
| Permeation | Proxylane | −2.78 ± 0.10 | −2.68 ± 0.17 | −2.57 ± 0.51 |
|  | Caffeine | 1.34 ± 0.35 | 1.33 ± 0.35 | 1.09 ± 0.62 |
|  | Minoxidil | −3.05 ± 1.64 | −3.06 ± 1.85 | −2.80 ± 1.70 |
|  | Compound X | −1.02 ± 0.69 | −1.07 ± 0.85 | −1.34 ± 1.30 |
|  | Oxybenzone | 0.74 ± 0.45 | 0.82 ± 0.60 | 0.58 ± 1.12 |
| Deposition | Proxylane | −1.66 ± 0.63 | −0.83 ± 0.76 | −1.16 ± 0.36 |
|  | Caffeine | − 0.71 ± 0.32 | −0.56 ± 0.33 | −1.24 ± 0.35 |
|  | Minoxidil | −1.54 ± 0.73 | −1.15 ± 0.80 | −1.27 ± 0.37 |
|  | Compound X | −0.39 ± 0.51 | −0.29 ± 0.68 | −0.19 ± 0.23 |
|  | Oxybenzone | 0.52 ± 0.25 | 0.70 ± 0.28 | 0.69 ± 0.18 |

Moreover, for each compound and operator pairs, **Figure S3** contains the *p*-values for the assessments of all sets of hypotheses for $H_{0}^{(P^{*})}$ and $H_{0}^{(D^{*})}$ using $t$-tests. Also in this case, since all *p*-values are greater than the corrected significance level $\alpha_{c}\approx0.003$, no null hypothesis can be rejected. Thus, there is no evidence of significant differences in experimental results at either the inter- or intra-laboratory level.


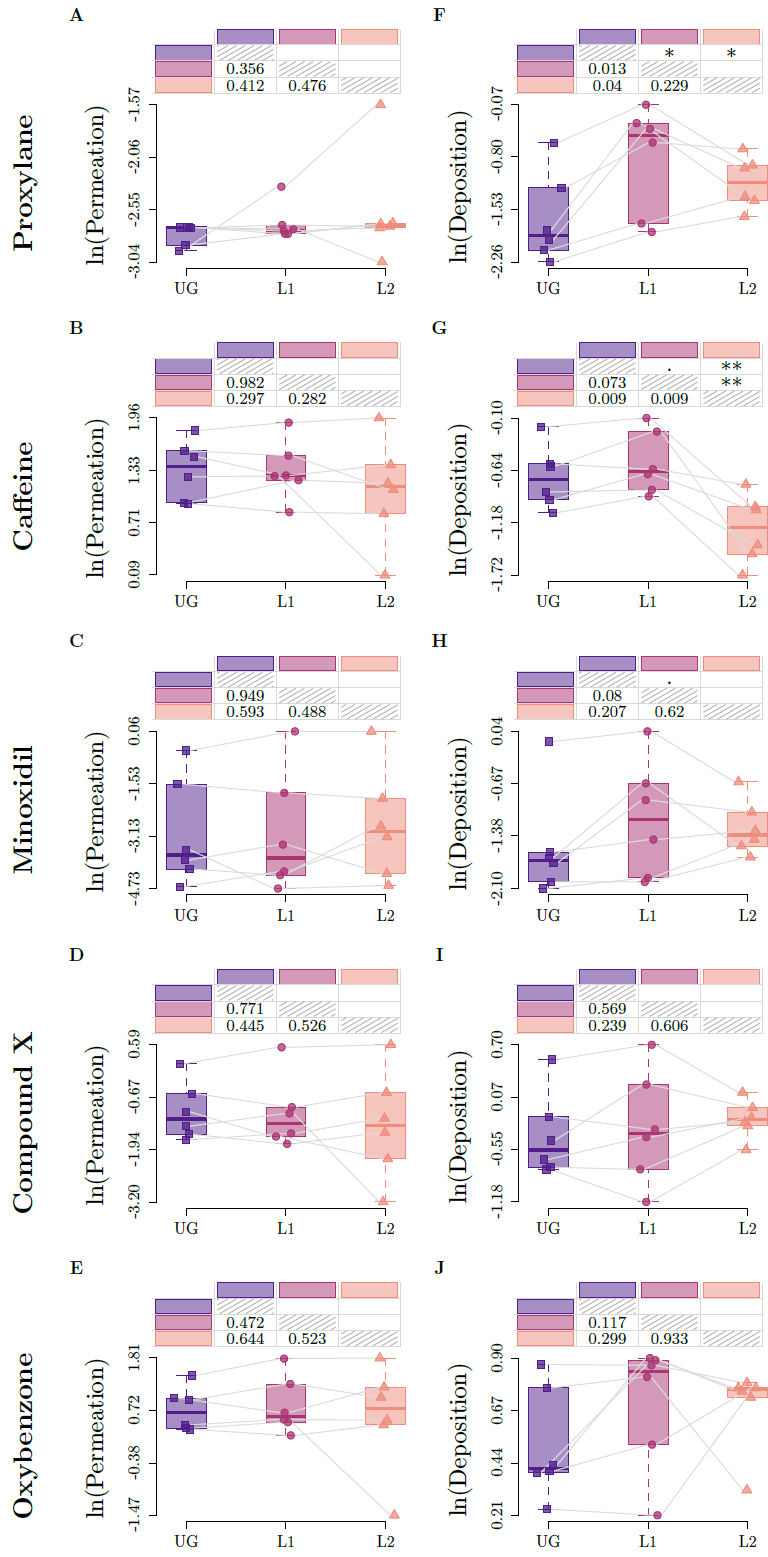


**Figure S3.** Log-transformed paired skin permeation (**A-E**) and total deposition (**F-J**) data for proxylane (**A, F**), caffeine (**B, G**), minoxidil (**C, H**), compound X (**D, I**), oxybenzone (**E, J**) generated by University of Geneva (UG), L’Oréal operator 1 (L1), and L’Oréal operator 2 (L2) for intra- and inter-laboratory validation ( Mean ± SD, n=6). For each pairwise comparison across the three individual operators, *p*-values obtained from paired, two-sided t-tests are reported, and tests are performed at the corrected significance level$\alpha_{c}=0.05/15\approx0.003$ ($\alpha_{c}=\alpha/T_{1}$, where $T_{1}=15$ accounting for 3 operator comparisons × 5 compounds). The symbols “.”, “*”, “**” and “***” indicate p-values respectively smaller than 0.1, 0.05, 0.01 and 0.001.


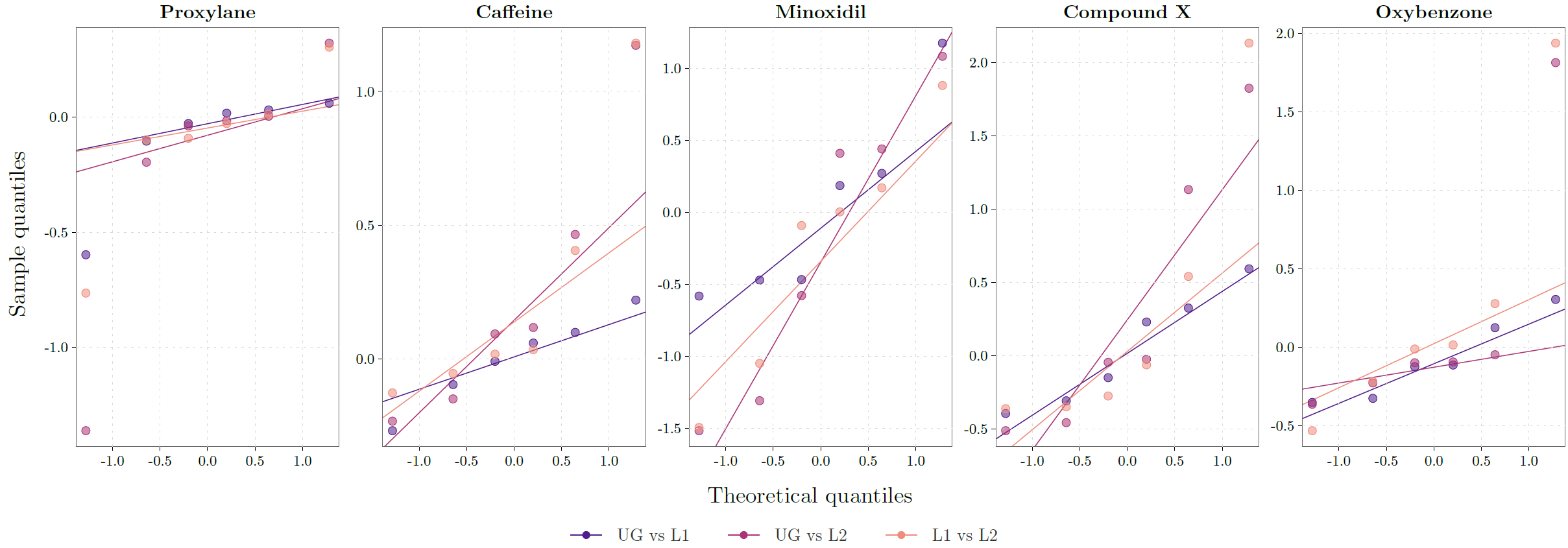


**Figure S4.** Normal Q-Q plots of the paired log-differences in skin permeation for each compound (proxylane, caffeine, minoxidil, compound X, oxybenzone; left to right), with the three pairwise operator comparisons (UG vs L1, UG vs L2, L1 vs L2; n=6) overlaid by colour.


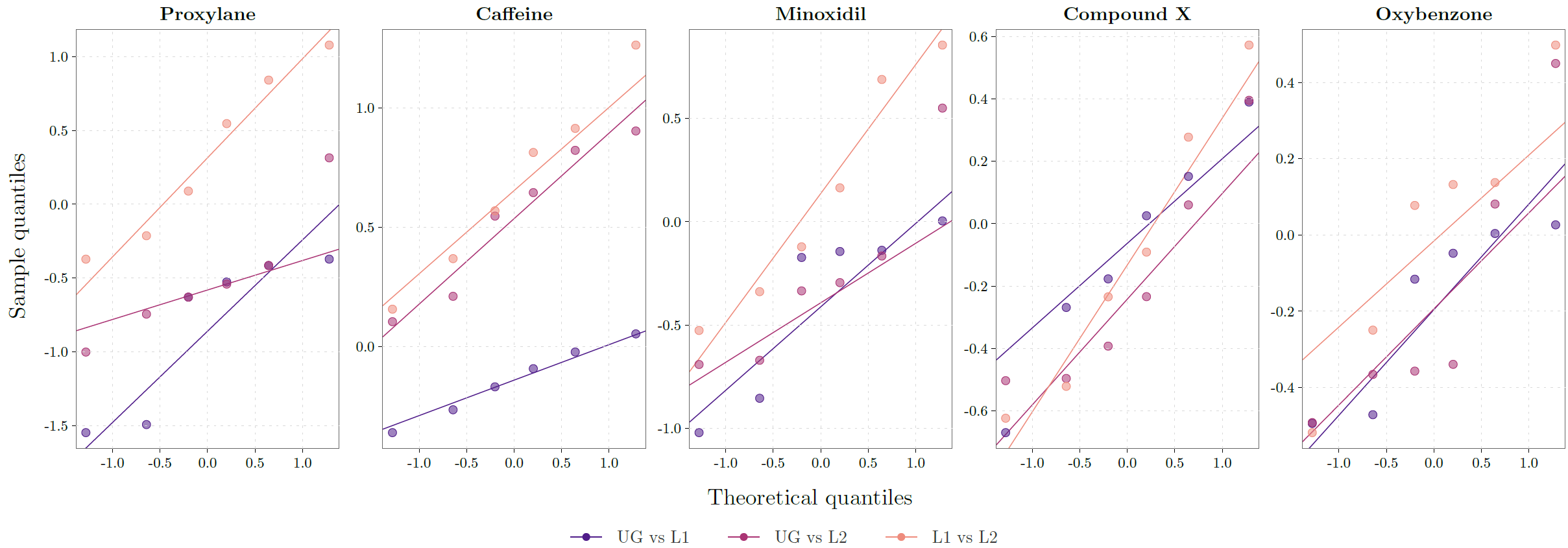


**Figure S5.** Normal Q-Q plots of the paired log-differences in total skin deposition for each compound (proxylane, caffeine, minoxidil, compound X, oxybenzone; left to right), with the three pairwise operator comparisons (UG vs L1, UG vs L2, L1 vs L2; n=6) overlaid by colour.


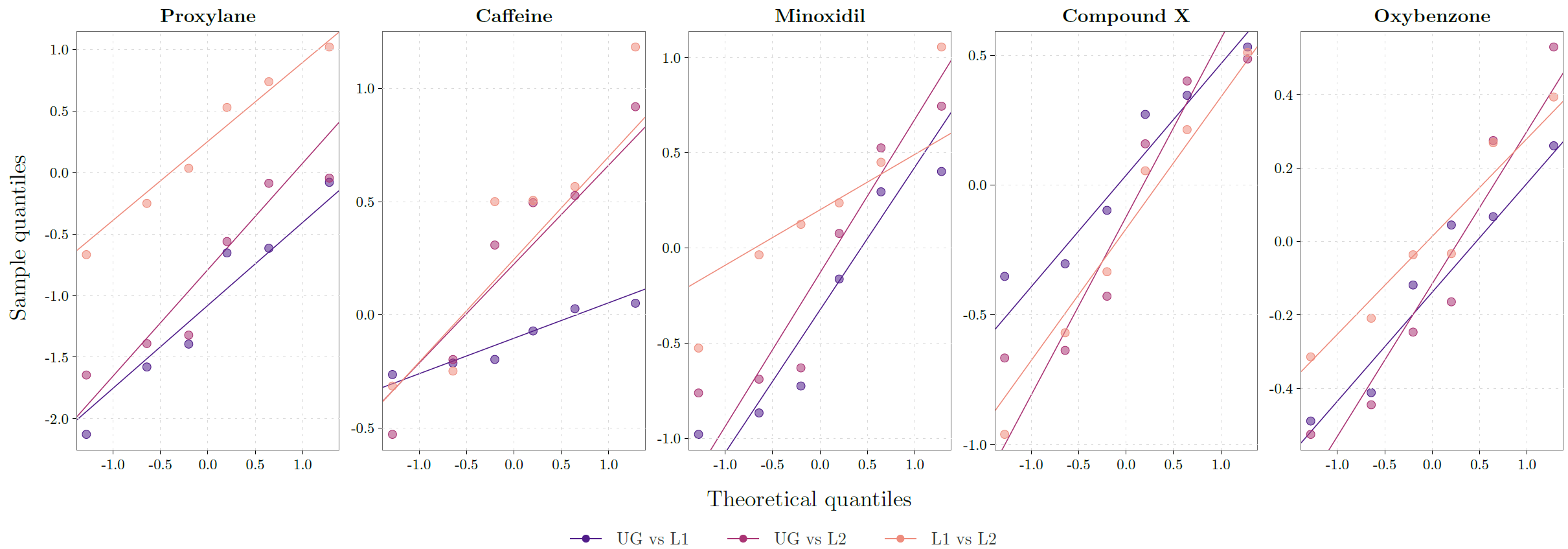


**Figure S6.** Normal Q-Q plots of the paired log-differences in cutaneous biodistribution (per-donor mean log-amount across skin depths) for each compound (proxylane, caffeine, minoxidil, compound X, oxybenzone; left to right), with the three pairwise operator comparisons (UG vs L1, UG vs L2, L1 vs L2; n=6) overlaid by colour.


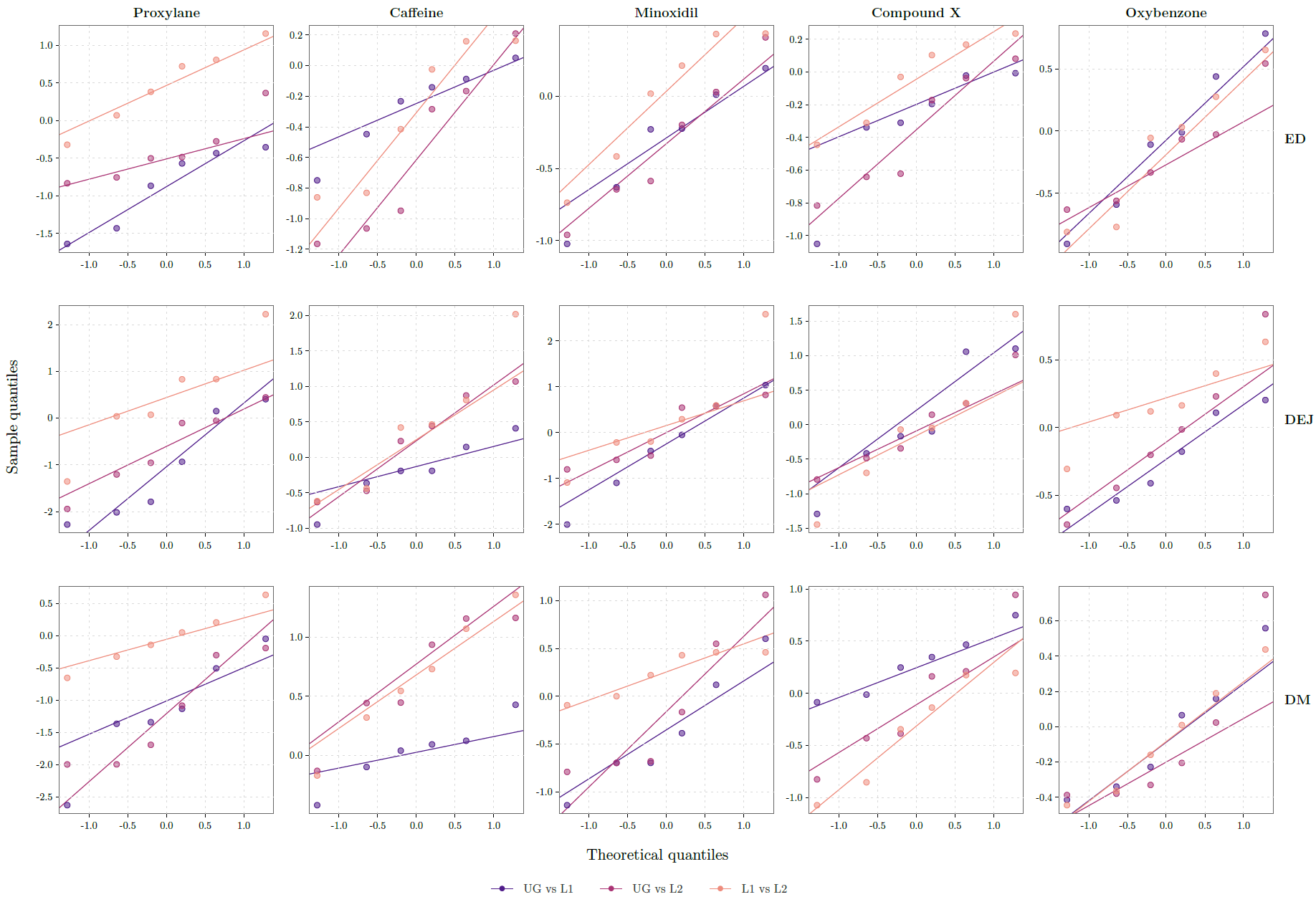


**Figure S7.** Normal Q-Q plots of the paired log-differences in cutaneous biodistribution by skin region (rows: ED, DEJ, DM) for each compound (proxylane, caffeine, minoxidil, compound X, oxybenzone; left to right), with the three pairwise operator comparisons (UG vs L1, UG vs L2, L1 vs L2; n=6) overlaid by colour.
